# Gait analysis in dogs with pelvic fractures treated conservatively using a pressure-sensing walkway

**DOI:** 10.1186/s13028-015-0158-3

**Published:** 2015-10-05

**Authors:** Flávia Gardilin Vassalo, Sheila Canevese Rahal, Felipe Stefan Agostinho, Maria Jaqueline Mamprim, Alessandra Melchert, Washington Takashi Kano, Luciane dos Reis Mesquita, Danuta Pulz Doiche

**Affiliations:** Department of Veterinary Surgery and Anesthesiology, School of Veterinary Medicine and Animal Science, Botucatu, SP Brazil; Department of Animal Reproduction and Veterinary Radiology, School of Veterinary Medicine and Animal Science, Botucatu, SP Brazil; Department of Clinic Veterinary, School of Veterinary Medicine and Animal Science, Botucatu, SP Brazil

**Keywords:** Dogs, Gait analysis, Fracture, Treatment, Kinetic

## Abstract

**Background:**

This study aimed to evaluate dogs with pelvic fractures and treated conservatively during locomotion on a pressure-sensing walkway. The hypothesis was that dogs may present changes in kinetic and temporospatial parameters because of the fractures, which may interfere with the symmetry index. Thirty dogs were selected and divided into two groups: Group 1—healthy group (n = 15) and Group 2—conservatively treated group (n = 15). The dogs were of similar body size. The body weight distribution percentages and symmetry indices of the peak vertical force, vertical impulse, stance time, swing time, percentage of stance time, and percentage of swing time of the hind limbs were evaluated.

**Results:**

In Group 2, the time interval between fracture occurrence and patient evaluation was between 4 and 87 months (mean of 20 months). Four dogs had lower percentage of body weight distribution on one of the hind limbs while three dogs had greater weight distributed toward both hind limbs. Four of these dogs had alterations in the temporospatial and/or kinetic symmetry indices.

**Conclusions:**

Dogs with pelvic fractures treated conservatively may present changes in percentage of body weight distribution and symmetry indices of the kinetic and temporospatial parameters. The conservative treatment can cause persistent abnormal gait.

## Background

Pelvic fractures comprise approximately 20–25 % of the fractures observed in small animals with a large percentage being due to motor-vehicle accidents [1–5]. Other causes include fights, falls, kicks, blunt trauma and gunshot wounds, in addition to stress and pathological fractures [2–6].

Around 75 % of the dogs and cats with pelvic fractures are able to recover without a surgical procedure [4]. Included in this category are the pelvic fractures in proper alignment or those comprising less vital portions of the pelvis, and when continuity of the pelvic canal remains intact [1, 3]. This is due to the extensive blood supply to the pelvic bones and heavy muscles surrounding the pelvis that aid in immobilizing the fractured segments [2, 3]. The perfect anatomical alignment of the fractures is not necessary for their consolidation or function [3]. However, conservative management requires rest of the restricted site for 2–8 weeks [6].

The criteria used to justify surgical intervention are displaced acetabular fractures, especially those involving the cranial 2/3 of acetabulum, severe narrowing of the pelvic canal, neurological changes, ipsilateral fractures of the ilium, ischium and pubis promoting an unstable hip joint, grossly displaced fragments, and other injuries that necessitate early support of the pelvic fractures [1, 3, 4, 6].

To determine the validity for both conservative and surgical treatment, objective methods of gait analysis are necessary [7, 8]. If the dog presents hind-limb lameness, in general, a load redistribution occurs. The affected limb has lower kinetic parameters that may be increased; for example in the contralateral hind limb and/or ipsilateral forelimb as a compensatory mechanism [9, 10]. Furthermore, the normal locomotion of the dog is considered symmetric, but the presence of neurological or orthopedic injuries may affect this symmetry [8]. A perfect symmetry is represented by an asymmetry index near 0 %. Thus, studies have been undertaken to establish appropriate cut-off points for the symmetry indices to identify lameness [9, 10].

Although the pressure-sensing walkway has been used in some orthopedic or neurologic conditions in dogs [11–14], apparently it has not been used to evaluate dogs with pelvic fracture. The pressure-sensing walkway offer several advantages, such as the possibility of collecting multiple variables and consecutive footfalls that reduces the number of recordings required to produce an adequate amount of data [15–17]. Thus, the present study aimed to evaluate kinetic and temporospatial parameters of dogs with pelvic fractures treated conservatively as presented by their movement on a pressure-sensing walkway. The hypothesis was that dogs may have kinetic and temporospatial changes related to the injury, which may interfere with the symmetry index.

## Methods

### Study population

This study was approved by the Institutional Ethics Committee (no 38/2013-CEUA). Thirty dogs were selected and divided into two groups. Group 1 included 15 clinically healthy dogs (nos. 1–15) and consisted of 12 females and 3 males, mean age of 3.9 years (SD = 2.1 years) without prior history of orthopaedic conditions while Group 2 included 15 dogs (nos. 16–30) and consisted of 9 females and 6 males, mean age of 6.0 years (SD = 3.3 years) that had pelvic fractures treated conservatively. The dogs in the two groups had similar morphometric measurements.

Age was not considered as inclusion criteria. The majority of the dogs were crossbred (n = 24), while the others were Dachshunds (n = 2), Yorkshire (n = 1), Pinscher (n = 1), Pug (n = 1) and Shih Tzu (n = 1) breeds. Body size was estimated on the basis of forelimb length (distance from dorsal scapular border to the ground), hind limb length (distance from greater trochanter to the ground), and body length (distance from cranial aspect of the scapulohumeral joint to the caudal aspect of the ischial tuberosity). The measurements were performed by using a tape.

The Group 1 dogs were considered to be clinically healthy based on complete physical and orthopedic examinations, and radiographic evaluation of the pelvis and hind limbs. Group 2 included dogs with pelvic fractures that had occurred at least 4 months prior, had not received any anti-inflammatory drugs or pain medications for at least 1 month, and had not received rehabilitation plan or exercise regimen. Exclusion criteria included presence of fractures in other bones, or musculoskeletal changes that could interfere with the analysis. Data regarding cause of injury, time of occurrence, and complications were obtained. Complete physical and orthopedic examinations based on Millis and Levine [18] were performed and included: lameness scores (0: normal; 1: intermittent lameness; 2: obvious lameness with weight-bearing; 3: severe lameness with weight-bearing; 4: intermittent lameness without weight-bearing; 5: non-use), presence of pain (slight, moderate, or severe), crepitation of the hip joint during manipulation and hip palpation, and proprioception of the hind limbs. Ventrodorsal hip-extended and lateral radiographic views of the pelvis were obtained to classify the fractures, and to evaluate secondary osteoarthritis related to pelvic fractures.

### Data collection

Immediately before data collection, the dogs were weighed on the same electronic scale. The dogs were submitted to gait analysis using a 1.951 mm × 447 mm pressure-sensitive walkway (Walkway^®^; Tekscan Inc, South Boston, MA, USA). Designated software (Walkway 7.0 software^®^) was used for acquisition and analysis of the temporospatial and kinetic data. Before data collection, each dog was familiarized with the environment and pressure-sensing walkway using practice trials. The dogs were guided on a leash by the same handler across the pressure-sensing walkway in a straight line. The velocity was maintained at 0.9–1.1 m/s, which was comfortable for the dogs, and the acceleration between—0.15 and 0.15 m/s^2^, both recorded by pressure-sensitive walkway system. An average of 25 trials was recorded, and five valid trials were analyzed for each dog. A trial was valid if the limbs had made contact with the surface of the walkway at least two times, without the dog turning the head.

The temporospatial parameters evaluated were gait cycle time (s), stance time (s), swing time (s), and stride length (m). The stance time percentage was established by (stance time/gait cycle time) × 100. The swing time percentage was established by (swing time/gait cycle time) × 100. The stride was determined by the distance between two consecutive ground contacts of the same limb. The kinetic parameters evaluated were peak vertical force (PVF) and vertical impulse (VI). The PVF and VI were normalized to the dog’s body weight (BW) and characterized by percentage of BW respectively as %BW and %BW × s. The percentage of BW distribution among the four limbs during gait was calculated as: (PVF of the limb/total PVF of the 4 limbs) × 100.

### Symmetry index

The symmetry indices between right hind limb (RH) and left hind limb (LH) for Group 1 was calculated for kinetic and temporospatial variables by the following formula: 200 [(RH − LH)/(RH + LH)] [19]. The same formula was used for Group 2. Negative values were multiplied by −1 before calculating the mean value of the parameters. A symmetry index (SI) of 0 indicated perfect symmetry. Cut-off values to distinguish between normal hind limbs (Group 1) and abnormal hind limbs (Group 2) were determined by SI of the hind limbs of the Group 1 dogs, as follows: average SI + 2 times the SD. Based on this cut-off value, the hind limbs of the Group 2 dogs were considered with or without alteration in the kinetic or temporospatial parameters.

### Statistical analysis

For comparisons of body mass and body size (forelimb length, hind limb length and body length) between the groups, the normality of data was checked by the Kolmogorov–Smirnov test, followed by the unpaired *t* test. The same tests were used to compare the kinetic and temporospatial parameters between the forelimbs and between hind limbs of Group 1 dogs. Differences were considered significant at *P* < 0.05.

## Results

The statistical analysis showed that dogs in Group 1 and Group 2 did not differ in body mass, length of the forelimbs, length of the hind limbs or body length (Table 1).

**Table 1:** Evaluation of body mass and body size of Group 1 and 2 dogs

	Body mass (Mean ± SD)	Forelimb length (Mean ± SD)	Hind limb length (Mean ± SD)	Body length (Mean ± SD)
Group 1	11.27 ± 6.36	39.79 ± 11.63	34.28 ± 9.25	44.78 ± 9.57
Group 2	9.87 ± 5.97	37.71 ± 9.96	30.34 ± 8.50	41.95 ± 1042
*P*-value	0.54	0.60	0.24	0.45

*SD* standard deviation

In Group 2, the mean time interval between fracture occurrence and patient evaluation was between 4 and 87 months (mean 20 months) (Table 2). As to the cause of injury, 93 % of cases were attributed to being hit by a motor vehicle, whereas 7 % were unknown. The most important complication associated with conservative treatment was constipation in one dog (No. 24) due to reduction in pelvic canal width. Signs of slight pain were observed on forced extension of the hip joint in four dogs (Nos. 20, 22, 23 and 28) and restriction of the hip joint in one dog (No. 26). Proprioceptive deficits or signs of fracture movement were not detected. At orthopedic examination, 26.7 % of the dogs had a lameness score of 1 versus 46.7 % presenting a score of 2; signs of lameness were not detected by visual analysis in 26.7 % of the dogs (Table 2).

**Table 2:** Radiologic diagnosis of the fracture site, indication for surgery or not, time between fracture and the study, pelvic narrowing, and subjective lameness scoring for Group 2 dogs

Case no.	Dog signalment	Fracture site	Indication for surgery	Time of fracture (months)	Pelvic narrowing	Lameness score
16	11.3 kg 2-years-old male crossbreed	Right ilial body	No	8	No	0
17	6.1 kg 2-years-old female crossbreed	Right ilial and ischial bodies, and acetabulum; left ilial body and ischiatic table; bilateral pubic body	Yes	4	Yes	2
18	15.5 kg 2-years-old female crossbreed	Right ilial body and ischiatic tuberosity; left ischial body; pubic symphysis separation	Yes	4	Yes	2
19	16.7 kg 9-years-old female crossbreed	Right ilial and ischial bodies, and acetabulum; left sacroiliac separation; bilateral pubic caudal ramus	Yes	87	Yes	2
20	3.5 kg 5-years-old male crossbreed	Right ischial body and pubic body	No	30	No	0
21	7.8 kg 6-years-old female crossbreed	Right acetabulum; left ischial body; bilateral pubic body	Yes	16	No	1
22	5.0 kg 7-years-old male crossbreed	Right pubic caudal ramus; left ilial body and acetabulum	Yes	13.5	No	1
23	3.3 kg 13-years-old male crossbreed	Right pubic cranial ramus, and ischial body; left pubic body; pubic symphysis separation	No	61.5	Yes	0
24	18.0 kg 8-years-old female crossbreed	Bilateral ilial body	Yes	4	Yes	1
25	23.3 kg 6-years-old female crossbreed	Right ischial body, pubic cranial ramus; left ischial body, pubic cranial ramus and acetabulum	Yes	4	Yes	2
26	6.7 kg 8-years-old female crossbreed	Left ilial and ischial bodies; bilateral pubic body; pubic symphysis separation	Yes	8	Yes	0
27	11.5 kg 10-years-old female crossbreed	Right ilial body	No	18	No	2
28	5.8 kg 5-years-old male crossbreed	Right ilial body and acetabulum; bilateral pubic cranial and caudal ramus; left ischial body fracture; left sacroiliac separation	Yes	7	Yes	2
29	7.2 kg 3-years-old female crossbreed	Right pubic body and acetabulum	Yes	22	No	2
30	6.5 kg 4-years-old male dachshund	Right ilial body, pubic body and acetabulum; left pubic cranial ramus	Yes	14	No	1

Radiologic diagnosis of the fractures and indication for surgery or not are described in Table 2. In 93 % of the cases, the fractures included more than one bone of the pelvis. Of all 15 dogs, 13 had displaced pelvic fractures, and 10 had fractures in three or more directions. The fractures were unilateral in five dogs, and bilateral in ten dogs. Narrowing of the pelvic canal was detected in eight dogs, but only one presented an occurrence of constipation (Table 2). All dogs with articular fracture had signs of osteoarthritis.

Dogs in Group 1 presented no significant differences between right and left forelimbs, and between right and left hind limbs for all variables. The SI values of hind limbs of the Group 1 are described in Table 3. These values were used for comparison of the dogs in Group 2 (Table 4). The dogs 18, 19, 20, 22, 23, 24, 25, 26, 28 and 29 showed kinetic and/or temporospatial changes (Table 4).

**Table 3:** Percentage of body weight (BW) distribution and symmetry indices of the kinetic data and temporospatial parameters of the hind limbs in Group 1 dogs (controls)

	% of distribution	Symmetry indices					
		Stance time (s)	Swing time (s)	% of stance	% of swing	PVF (%BW)	VI (%BW*s)
Mean ± SD	19.89 ± 1.80	5.29 ± 4.21	4.24 ± 3.30	4.77 ± 3.29	4.33 ± 3.67	6.59 ± 4.81	6.67 ± 4.50
<cutoff value	16.28	–	–	–	–		
>cutoff value	23.50	13.70	10.84	11.35	11.68	16.21	15.67

**Table 4:** Fracture classification, symmetry indices of the kinetic data and temporospatial parameters, and percentage of body weight (BW) distribution of the hind limbs in Group 2 dogs based on reference values obtained from Group 1 dogs

Case no.	Fracture classification	Symmetry indices						% of BW distribution of the right hind limb	% of BW distribution of the left hind limb
		Stance time (s)	Swing time (s)	% of stance	% of swing	PVF (%BW)	VI (%BW × s)		
16	Unilateral	0.53	2.76	0.08	2.14	8.89	6.32	17.31	18.92
17	Bilateral	9.16	4.17	3.17	1.83	11.15	3.19	18.01	20.14
18	Bilateral	13.33	4.14	10.78	6.70	12.73	*23.40*	*16.02*	18.20
19	Bilateral	*26.49*	*22.82*	*24.89*	*24.42*	*20.61*	*46.60*	*14.88*	18.29
20	Unilateral	*14.00*	3.80	*11.52*	6.29	6.22	2.88	17.25	18.36
21	Bilateral	5.24	7.19	10.49	1.93	10.98	3.96	21.00	18.82
22	Bilateral	10.53	7.41	*12.44*	5.49	9.36	3.54	22.54	*24.75*
23	Bilateral	5.71	2.88	6.76	1.83	*25.88*	*30.14*	*23.92*	18.44
24	Bilateral	10.06	9.38	9.40	10.03	*19.22*	*23.33*	18.96	22.99
25	Bilateral	7.09	8.58	6.25	9.42	*32.37*	*30.72*	20.81	*15.01*
26	Bilateral	0.67	1.75	0.49	2.92	4.32	4.98	*25.60*	*26.73*
27	Unilateral	0.00	3.15	2.26	0.89	8.10	7.10	20.46	18.86
28	Bilateral	11.57	9.00	*11.94*	8.63	*21.53*	*24.49*	21.33	17.18
29	Unilateral	*32.68*	*15.25*	*23.49*	*24.53*	*26.05*	*42.86*	*14.72*	19.12
30	Bilateral	4.20	7.53	5.43	2.09	12.04	15.19	19.73	17.49

Values exceeding the cutoff values are shown in italics

## Discussion

In the present study, 93 % of cases presented pelvic fractures caused by motor vehicle accidents, also the most common cause cited in prior studies [1–5]. The severity of the fracture displacement, location of the fracture and degree of pelvic canal narrowing are factors that must be considered in treatment selection [6, 20]. Surgical treatment was indicated in 11 dogs (73 %), according to the criteria reported previously [5, 6, 20]. However, factors such as limited financial resources of the owner, time interval between fracture and patient evaluation, and presence of severe injuries to other organs made the surgical procedure unfeasible. As reported previously, muscle contraction and fibrosis hamper reduction of the fracture, and may cause iatrogenic surgical trauma [5, 6]. Therefore, after 7–10 days, other methods may be more appropriate than the primary repair of the fracture [5].

Cited complications of conservative treatment have included mal-union or pelvic canal narrowing that can result in constant or intermittent constipation, especially in cases of pelvic narrowing of 50 % or more [3, 5]. Only one dog (No. 24) showed constipation, although pelvic narrowing was present in 53 % of the dogs. Although medial narrowing of the pelvis was observed, the dorsoventral displacement of a hemipelvis relative to the other allowed accommodation of the rectum.

In addition, malalignment and/or fracture instability may cause limited movement of the hip joint [6], as observed unilaterally in one dog (No. 26) due to severe hemipelvic dislocation. On the other hand, malalignment can induce pain, which was induced in 27 % of dogs on forced extension of the hip joint. In addition, signs of bone healing was found by radiographic examination in 30 % of the cases. Movement of the fracture was not detectable by physical examination in the other cases, probably due to fibrosis around the fracture. Usually contraction of the pelvic muscles stabilizes the fractures not internally displaced [21].

Although the morphometric measurements (lengths of the limbs and body length) did not differ statistically between the groups, as the population heterogeneity required that the evaluations of kinetic data and temporal-spatial parameters between groups were done using SI, as suggested in other studies [9]. The soundness of Group 1 dogs was confirmed by absence of significant differences in kinetic data and temporospatial parameters between forelimbs or between hind limbs. In addition, the percentage of BW distribution was approximately 30 % on each forelimb and 20 % on each hind limb, consistent with a previous description of clinically healthy dogs in locomotion over a pressure-sensing walkway [22].

With respect to Group 2, the four dogs that showed a lower percentage of BW distribution in one of the hind limbs had a lameness score of 2. Three of these dogs (Nos. 18, 19, and 25) had bilateral fractures with more severe radiographic changes on the side where less weight was distributed, while dog No. 29 had pelvis unilateral fracture with acetabular involvement. In a study that induced lameness in the right hind limb, it was observed that the dogs walking and trotting on an instrumented treadmill had all parameters decreased in this limb, and the center of mass was shifted to the contralateral side and cranio-caudally to the side opposite to the right hind limb [10].

Two dogs in the present study showed higher percentage of BW distribution in one of the hind limbs (Nos. 22, 23), and presented respective lameness scores of 1 and 0. Both had bilateral fractures, but more severe on one side; one with acetabular involvement (No. 22) and the other with fracture of the body of the ischium (No. 23), which justifies BW redistribution to the less affected side. On the other hand, dog No. 26, whose lameness score was 0, showed higher BW redistribution to both hind limbs, suggesting alterations in forelimbs. Thus, the lameness score determined in the present study did not always correspond with kinetic data. Similarly, in a study of dogs undergoing unilateral tibial osteotomy treated with external fixator, it was observed that subjective lameness scoring scales did not reproduce the data obtained by force platform analysis [23].

Five dogs showed changes in SI of the temporospatial parameters (Nos. 19, 20, 22, 28, and 29). In relation to pelvic fractures, three dogs had bilateral fractures (Nos. 19, 22, and 28) and two unilateral (Nos. 20 and 29). However, SI must be evaluated together with the other data, since low values of the temporospatial variables may suggest asymmetry, which may not be a true representation [24]. For example, SI of the temporospatial parameters of the dogs Nos. 20 and 22 showed asymmetry in percentage of stance time, but not in percentage of swing time, suggesting capture artefact. In addition, SI of the PVF and VI must also be evaluated together with the percentage of body weight distribution, especially in bilateral fractures. For example, the dog No. 26 had PVF and VI without SI alteration, but the percentage of BW distribution was higher in both hind limbs. Thus, only the dog Nos. 19 and 29 showed true changes in SI of the temporospatial parameters. These parameters are suggestive of inadequate function of the limbs, since the ratio of duration between the time when the foot is on the ground (stance time) and the time when the foot is off the ground (swing time) [13, 25, 26] was not maintained. In addition, both dogs showed changes in kinetic data.

Although 75 % of dogs with pelvic fractures are able to recover without a surgical procedure [4], analyzing the data obtained in the present study, 46.7 % of the dogs had some abnormality of percentage of BW distribution, suggesting that the conservative treatment may not be adequate if a normal biomechanical performance is desired after treatment.

A limitation that needs to be considered when interpreting the findings of the present study is the small sample size based on fracture type. Another limitation relates to a retrospective nature of the study as treatment was not determined exclusively by the fracture type.

## Conclusions

Dogs with pelvic fractures treated conservatively may present changes in percentage of BW distribution and SI of the kinetic and temporospatial parameters. The conservative treatment can cause persistent abnormal gait.

## Abbreviations

BW: body weight
LH: left hind limb
PVF: peak vertical force
RL: right hind limb
SD: standard deviation
SI: symmetry index
VI: vertical impulse
